# *Akkermansia muciniphila* Enhances the Antitumor Effect of Cisplatin in Lewis Lung Cancer Mice

**DOI:** 10.1155/2020/2969287

**Published:** 2020-08-07

**Authors:** Zhuo Chen, Xiang Qian, Shasha Chen, Xiaoxuan Fu, Guanjun Ma, Aiqin Zhang

**Affiliations:** ^1^Institute of Cancer and Basic Medicine (ICBM), Zhejiang Cancer Hospital, Cancer Hospital of the University of Chinese Academy of Sciences, No. 38, Guangji Road, Hangzhou, 310022 Zhejiang, China; ^2^Department of Traditional Chinese Medicine, Taizhou Cancer Hospital, No. 50, Zhenxin Road, Taizhou, 318000 Zhejiang, China; ^3^Second Clinical Medical College, Zhejiang Chinese Medical University, No. 548, Binwen Road, Hangzhou, 310053 Zhejiang, China

## Abstract

Recently, intestinal flora plays a vital role in the occurrence and development of tumors and there is link between cancer immunotherapy and Akkermansia muciniphila (Akk). However, the therapeutic efficacy of Akk in lung cancer remained unclear. Hence, our study is aimed at investigating the antitumor effects of cisplatin (CDDP) combined with Akk on lung cancer. Using the murine lung cancer model by subcutaneously inoculating Lewis lung cancer model, 50 mice were divided into five groups: normal, model, CDDP, CDDP+Akk, and CDDP+antibiotics. After treatment within 5 weeks, compared with the model group, the administered group improved the changes of tumor pathomorphology. Compared with the CDDP group, CDDP combining with Akk slowed down the growth of tumor volume, downregulated the levels of ki-67, p53, and factor-associated suicide (Fas) ligand proteins and upregulated Fas proteins, increased the levels of interferon-*γ*, interleukin-6, and tumor necrosis factor-*α*, and suppressed the expression of CD4^+^CD25^+^Foxp3^+^ Treg in mouse peripheral blood and spleen. In addition, transcriptome analysis indicated that Akk combining with CDDP increased obviously the levels of IFI27l2 and IGFBP7 and was related to those pathways including the cytokine-cytokine receptor interaction, Th17 cell differentiation, FOXO, JAK-STAT, and PI3K-Akt signaling pathways. These results suggested that the therapeutic efficacy of the combined treatment of Akk and CDDP was superior to the only CDDP treatment, which could enhance immune regulation and would be a promising strategy for the treatment of lung cancer.

## 1. Introduction

Lung cancer is the most frequent cancer and the main reason of cancer-related death worldwide; the majority of which are diagnosed at late stages, and the long-term survival rates of patients remain poor [1]. Clinical applications of cancer immunotherapies, radio therapies, gene therapies, or target-oriented therapies have been suggested as potential approaches to lung cancer treatment. However, due to the considerable toxicity and in efficiency of these treatments, it remains unsatisfactory to the therapeutic outcome of standard radiotherapy and chemotherapy in some patients with an advanced stage of or locally recurring lung cancer [2].

The involvement of microorganisms in cancer has been increasingly accepted. It has been estimated that microorganisms account jointly for approximately 20% of all cancers in the worldwide scale. Recently, the microbial ecology in different types of tumors, as well as the roles of microorganisms in tumor formation, development, and response to treatments, has constantly emerging a new understanding with advances of metagenomics and bioinformatics [3]. Meanwhile, it was also underlined the importance of host-microbial and intermicrobial interactions in the cancer microbiota. Current hot research not only revolutionized our understanding of cancer biology but also explored fresh possibilities for cancer prevention, diagnosis, prognostication, and treatment [4]. The human gut is perhaps one of the most complex networks in the body and is colonized by trillions of microorganisms including bacteria, archaea, fungi, protists, and viruses, among which bacteria are the major inhabitants [5]. Tanoue et al. isolated a consortium of 11 bacterial strains from healthy human donor feces that is capable of robustly inducing interferon-gamma- (IFN-*γ*-) producing CD8 T cells in the intestine. Colonization of mice with the 11-strain mixture enhances both host resistance against Listeria monocytogenes infection and the therapeutic efficacy of immune checkpoint inhibitors (ICIs) in syngeneic tumor models [6].

The lung is a mucosal tissue colonized by a diverse bacterial community, and the study on microecology is in its infancy particularly in the field of lung cancer. Lung cancer is closely associated with chronic inflammation. It was reported that commensal microbiota promote lung cancer development. In practical terms, commensal bacteria fuelled interleukin-1*β* (IL-1*β*) and IL-23 production from myeloid cells, inducing proliferation and activation of lung-resident Vgamma6(+)Vdelta1(+) gamma delta T cells, and then those cells produced IL-17 and other operating molecules to promote inflammation and tumor cell proliferation [7]. Existing researches potentially point to a link local microbiota-immune crosstalk to lung tumor development and thereby define pivotal cellular and molecular mediators that may serve as valid targets in lung cancer intervention.

*Akkermansia muciniphila* (Akk), one of the most dominant bacteria, exists in the mucus layer of the intestinal tract, constituting 1–4% of the total bacterial cells in the healthy adult feces [8]. Accumulating research evidences uncovered the beneficial effects of Akk in host [9–11]. It was reported that differential expression of tumor-associated genes and altered gut microbiome with decreased Akk afford a tumor-preventive microenvironment in intestinal epithelial Pten-deficient mice [12]. Fecal Akk is associated with body composition and microbiota diversity in overweight and obese women with breast cancer participating in a presurgical weight loss trial (Fruge et al. 2018). ICIs have achieved significant efficacy in the treatment of advanced lung cancer. ICIs would attract sustained clinical responses in a sizable minority of tumor sufferer through regulating the PD-1/PD-L1 axis, which was correlated with the relative abundance of Akk. Interestingly, Routy et al. revealed that oral supplementation with Akk after fecal microbiota transplantation from cancerous person who nonresponded to ICIs repaired the blockage effectiveness of PD-1 in an IL-12-dependent manner by adding the recruitment of CCR9(+)CXCR3(+)CD4(+) T lymphocytes into mouse tumor beds [13]. The precise relationship between Akk and antitumor effect in vivo is still poorly understood. Therefore, the mechanism of Akk in tumor immune microenvironment deserves further exploration.

The goal of this study was to investigate whether Akk enhances the antitumor effect of cisplatin (cis-diamminedichloroplatinum, CDDP), as the first line of the treatment in lung cancer or not. CDDP and Akk were combined to intervene in Lewis lung cancer mice. In vivo imaging was used to evaluate tumor size, and distribution and pathomorphologic changes were determined. Transcriptome sequencing was used to screen differentially expressed genes in the CDDP treatment group and the CDDP+Akk group, as well as the signaling pathways related to these differentially expressed genes. The levels of tumor marker proteins ki-67, p53, Fas/FasL, and immune cytokines and the proportion of CD4^+^CD25^+^Foxp3^+^ Treg cells were further detected.

## 2. Materials and Methods

### 2.1. Bacterial Strains and Growth Conditions

Akk (ATCC BAA-835) was cultured in sterilized brain heart infusion broth that was prepared with II mucins (Sigma), brain extract powder (OXOID), and deionized water, by the high pressure steam sterilization, and then at 37°C in an airtight pot called MiniMACS anaerobic incubator (Don Whitley Scientific) for approximately 48 h to reach a late exponential growth phase under strict anaerobic conditions. Cultures were centrifuged at 11,500 *g* for 10 min and washed three times with sterile phosphate-buffered saline (PBS). Then, the bacterial cells were resuspended with sterile PBS to 10^8^ colony-forming units (cfus)/0.2 mL and were deposited on ice immediately before administering to each mouse by gavage.

### 2.2. Cell Culture

Mouse Lewis lung cancer cell line was purchased from Shanghai Institute of Life Science, Chinese Academy of Sciences (Shanghai, China), subsequently cultured in Dulbecco's modified Eagle's medium (Thermo Fisher Scientific, USA) supplemented with 10% fetal bovine serum (Gibco, USA) in CO_2_ culture chamber (37°C, 5% CO_2_). After the growth density reached 70~80%, the cells were digested with 0.25% trypsin and subculturing.

.

### 2.3. Establishment of the Mouse Model and Treatment

50 female C57BL/6 mice were purchased from Shanghai Sipubikai Experimental Animal Co., Ltd. (Shanghai, China) and maintained in specific pathogen-free grade conditions until reaching an age of 4 weeks and a weight of 18–22 g (animal license number: SCXK; 2013-0016). A total of 50 mice were randomly divided into five groups: normal group, model group, CDDP treatment group, CDDP+antibiotics (ABx) group, and CDDP+Akk group. Lewis lung cancer cells (~4 × 10^7^/mL) were subcutaneously injected into the caudal vein of each mouse except for the normal group to establish the tumor models with hematogenous metastasis. CDDP (Sigma-Aldrich) was dissolved in physiological solution to make a stock solution of 1 mg/mL. Antibiotics were administered in drinking water in the following concentrations: ampicillin (1 g/L), vancomycin (0.5 g/L), neomycin trisulfate (1 g/L), and metronidazole (1 g/L). The CDDP+Akk group mice were administrated 10^8^ cfu Akk suspended in 0.2 mL sterile anaerobic PBS by oral gavage per day and intraperitoneal injection of CDDP (5 mg/kg) (once every two days); the CDDP group mice were intraperitoneal injection of CDDP (5 mg/kg). Intraperitoneal injection of CDDP (5 mg/kg) was combined with a cocktail of antibiotics in the CDDP+ABx group, while mice in the model and normal groups were given an equivalent volume of sterile anaerobic PBS instead. Treatment was continued for five weeks; the body weights of the mice were measured weekly.

All mice were maintained in a temperature (22 ± 2°C)- and humidity (50-60%)-controlled environment under a 12 h light–dark cycle. Mice in each group ate and drank freely. All experiments were approved by the Local Committee of Animal Use and Protection and were performed in accordance with criteria of *Guide for the Care and Use of Laboratory Animals*.

### 2.4. Determination of Tumor Growth

The tumor volume of mice was observed five weeks post inoculation with vernier caliper (8015, SANTO). After the mean tumor volume of all mice inoculated with tumor reached 100 mm^3^, the volume was measured at a twice-weekly frequency and determined to establish a tumor growth curve. Tumor volumes were determined using the following formula: tumor volume (cm^3^) = 1/2∗(length [mm]∗width^2^ [mm]). Before the end of the experiment, the tumor status of the mice was observed by small animal in vivo imaging (IVIS Lumina LT, Caliper). The rats were intraperitoneally injected with 150 mg/kg fluorescein potassium and metabolized for 10 min. After being anesthetized with isoflurane gas, the rats were put into the imaging chamber and the automatic exposure time was set to dynamically observe tumor growth.

### 2.5. Observation of Tumor Histopathology by H&E Staining

After the mice were sacrificed, tumor samples from each group were harvested, fixed in 10% formaldehyde overnight, then embedded in paraffin, sectioned (3 *μ*m thick), and stained with hematoxylin and eosin (H&E) for general histology. The samples were observed and photographed under the microscope (Eclipse TI-SR, Nikon, Japan).

### 2.6. RNA Isolation, Purification, and Quality Control

After the mice were sacrificed, peripheral lymphatic tissue was taken from the CDDP and CDDP+Akk mice, and the surface adipose tissue was removed from the DEPC water. The total RNA was isolated and purified by TRIzol (Invitrogen, Carlsbad, CA, USA) method. Then, NanoDrop 1000 (NanoDrop, Wilmington, DE, USA) was used to control the quantity and purity of total RNA. The integrity of RNA was detected by Agilent 2100, and RIN number > 7.0 used as the qualified standard.

### 2.7. Preparation and Sequencing of cDNA Library

5 g of total RNA was taken out, and mRNA containing PolyA was specifically captured using oligo (dT) magnetic beads through two rounds of purification. The captured mRNA was segmented by divalent cation at high temperature. The fragmented RNA was synthesized into cDNA by reverse transcriptase. E. coli DNA polymerase I was used for two-strand synthesis with RNase H. These double strands of DNA and RNA are converted into DNA double strands. Meanwhile, DUTP was added into the two-strand to complement the ends of double-strand DNA into flat ends. In addition, A base is added at each end, so that it can be connected with the connector with T base at the end, and then the fragment size is screened and purified by magnetic beads. The second chain was digested with UDG enzyme, and then PCR was used to form a library with fragment size of 300 bp (±50 bp). Finally, Illumina Hiseq 4000 (LC Bio, China) was sequenced according to standard operation with a reading length of 150 bp.

### 2.8. Bioinformatics Analysis

Using Illumina 4000 sequence analysis platform, cDNA library was constructed and sequenced from mixed RNA from mouse paracancer lymphoid tissue samples. HISAT software package was used to compare the readings of the CDDP treatment group samples and the CDDP+Akk group samples with UCSC(http://genome.ucsc.edu/), and then the readings were mapped to the reference genome. The graph readings for each sample were assembled using StringTie. A Perl script is then used to combine all transcripts in the sample to reconstruct a comprehensive transcriptome. StringTie and Edger were used to estimate the expression levels of all transcripts. Finally, “log2(fold change) > 1 or log2(fold change) < −1 and *P* < 0.05” was used as the screening condition for genes with statistical differences. Immune-related genes were screened again in the differential genes, and the acquired immune-related differential genes were analyzed by cluster analysis, GO (Gene Ontology) enrichment, and KEGG pathway annotation.

### 2.9. Determination of ki-67, p53, Fas, and FasL Protein by Western Blotting

After the mice were sacrificed, tumor tissue (about 100 mg) from each mouse was homogenized in cold lysis buffer and subsequently total proteins were extracted. Protein concentrations were detected via BCA kit (Beyotime Institute of Biotechnology, Shanghai, China). Protein samples (50 *μ*g) were separated via 10% SDS-PAGE and subsequently transferred to the PVDF membranes (Millipore, Billerica, MA, USA). Following this, the membranes were blocked for 1 h and subsequently incubated with the antibodies (dilution ratio 1 : 1000) at 4°C over the night: ki-67 (AF0198, affinity), p53 (AF0879, affinity), Fas (AF5342,affinity), and FasL (AF0157,affinity). GAPDH (10494-1-AP. Proteintech, dilution ratio 1 : 5000) was used as an endogenous reference. The membranes were washed and then incubated with horseradish peroxidase- (HRP-) labeled anti rabbit (7074, CST) or mouse (7076, CST) secondary antibodies (dilution ratio 1 : 3000) at room temperature for 2 h. The membranes were washed and developed by supersensitive chemiluminescence using Mini-PROTEAN gel imaging system (Bio-Rad, USA). ImageJ software (National Institutes of Health, Bethesda, Maryland, USA) was used to quantify each band area and integrated density in the blots.

### 2.10. Determination of IFN-*γ*, IL-6, IL-10, and TNF-*α* Levels in Tumor by ELISA Assay

Before mice in each group were sacrificed, serum samples from retroorbital were collected to determine the concentrations of IFN-*γ*, IL-6, IL-10, and TNF-*α* using the corresponding standard quantitative enzyme-linked immunosorbent assay (ELISA) kits. The absorption value of each group of samples was measured by a microplate reader (CMax Plus, Thermo Scientific, USA).

### 2.11. Detection of Ratio of CD4^+^CD25^+^Foxp3^+^ Treg (Regulatory T Cell) Cells in Mouse Peripheral Blood and Spleen Mononuclear Cells by Flow Cytometry

Mononuclear cells were isolated after the use of erythrocyte lysate to lyse anticoagulant blood and spleen tissue fluid in experimental mice. Surface molecules were stained using a standard procedure, with a fluorescein isothiocyanate (FITC)-CD4 antibody (lot100406, BioLegend, USA) and an phycoerythrin conjugated (PE)-CD25 antibody (lot102008, BioLegend, USA), and incubated at 4°C for 15 min. After being washed 2 times with flow cytometry staining buffer, the cells were immobilized by rupture of the membrane with permeabilization working solution. Then, intracells were stained with an Alexa Fluor 647 FOXP3 antibody (lot320013, BioLegend, USA) and incubated at 4°C for 30 min. Finally, the cells were washed, resuspended, and analyzed with flow cytometry (Accuri C6, BD, USA). The proportion of positive cells was determined using WinMDI version 2.8 software (The Scripps Institute, USA).

### 2.12. Statistical Analysis

All data were presented as the means ± standard deviation (SD) and analysed using SPSS software version 17.0 (Stanford University, San Francisco, California, USA). Differences between the control and the treatment groups were determined using one-way analysis of variance and followed by the least significant difference (LSD) multiple range tests. The values of *P* < 0.05 and *P* < 0.01 were used as the criterion for statistical significance.

## 3. Results

### 3.1. Effect of Akk on Tumor Volume Combined with CDDP

Firstly, the weight and tumor volume of mice were recorded during treatment. As shown in Table 1, the weight of mice in the given drug groups was lower than the normal and model group during the experiment, especially the CDDP group and CDDP+ABx group (*P* < 0.05).As shown in Table 2, compared with the model group, the tumor volume of the administration group decreased to a certain extent. Starting from the 4 weeks, the tumor volume in the CDDP group or the CDDP+Akk group was less than that of the model group (*P* < 0.05, *P* < 0.01). Compared with the CDDP group, tumor volume in the CDDP+Akk group showed a decreasing trend, hinting that the efficacy of Akk combined with CDDP was better than that with CDDP alone. Tumor volume in the CDDP+ABx group was decreased, but was not as good as that of the CDDP group and showed no significant difference (*P* > 0.05).

In addition, the growth of lung cancer in mice was observed by in vivo imaging.

**Figure 1:**
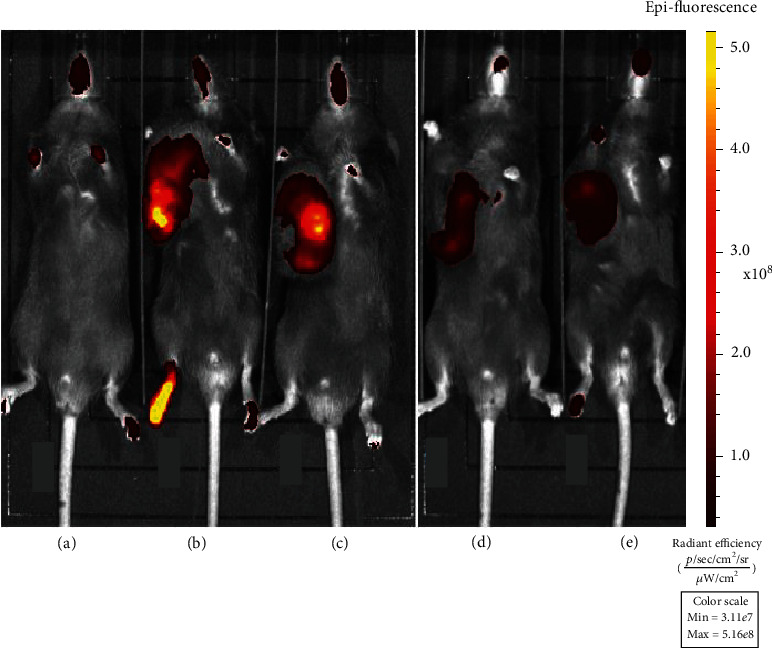
Observation in vivo imaging of the effect of CDDP combined with Akk on the growth of lung cancer in mice. (a) Normal. (b) Model. (c) CDDP. (d) CDDP+Akk. (e) CDDP+ABx.

In the normal group, the area of the red part in mice was mostly absent (Figure 1). And the area of the red part in mice from the model group was large and the color was bright. Compared with the model group, the area of the red part was slightly smaller in the CDDP group. The red color in the CDDP+Akk group and the CDDP+ABx group became darker, and the area of the CDDP+Akk group decreased significantly.

### 3.2. Effect of Akk on Tumor Pathomorphology Combined with CDDP

**Figure 2:**
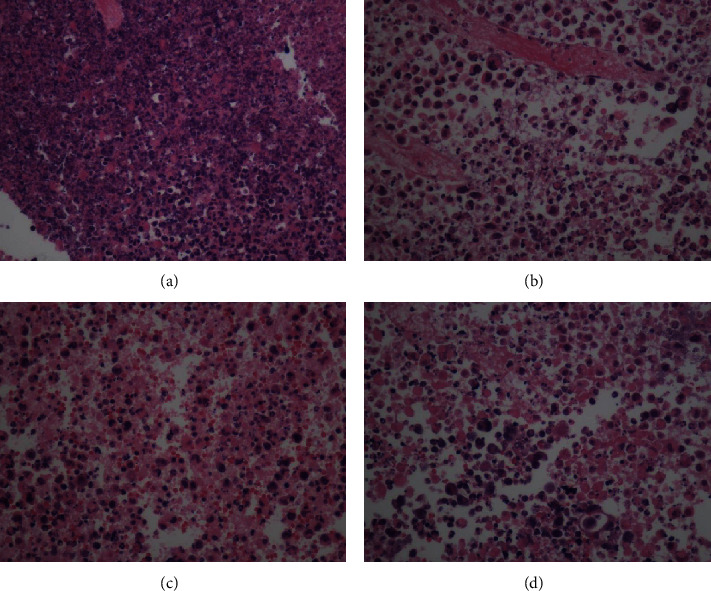
Effect of CDDP combined with Akk on tumor pathology in lung cancer mice. The pathological picture of the model control group (a), the CDDP group (b), the CDDP+Akk group (c), and the CDDP ABx group (d). Lung tissues were fixed, stained with H&E solution, and observed under a microscope of 400 magnifications.

Pathomorphology of tumor tissues in lung cancer mice was observed by H&E staining. As shown in Figure 2, in the model control group, the tumor tissue cells were clear in contour and presented flaky or nest-like distribution. The cells grew vigorously without necrosis and the nuclei were deeply stained. The tumor tissue cells in the administration group all showed degeneration and necrosis of different degrees. Meanwhile, the vascular components in the interstitium of the administration group were significantly reduced. The matrix and stroma were reduced in the CDDP and CDDP+ABx groups, and the tumor tissues showed nuclear condensation and patchy necrosis. The tumor tissues in the CDDP+Akk group contained nuclear condensation, large fragments of cell disintegration, and necrosis.

### 3.3. Effect of Akk on Immune-Related Differential Genes and Pathways Combined with CDDP in Tumor Tissues

**Figure 3:**
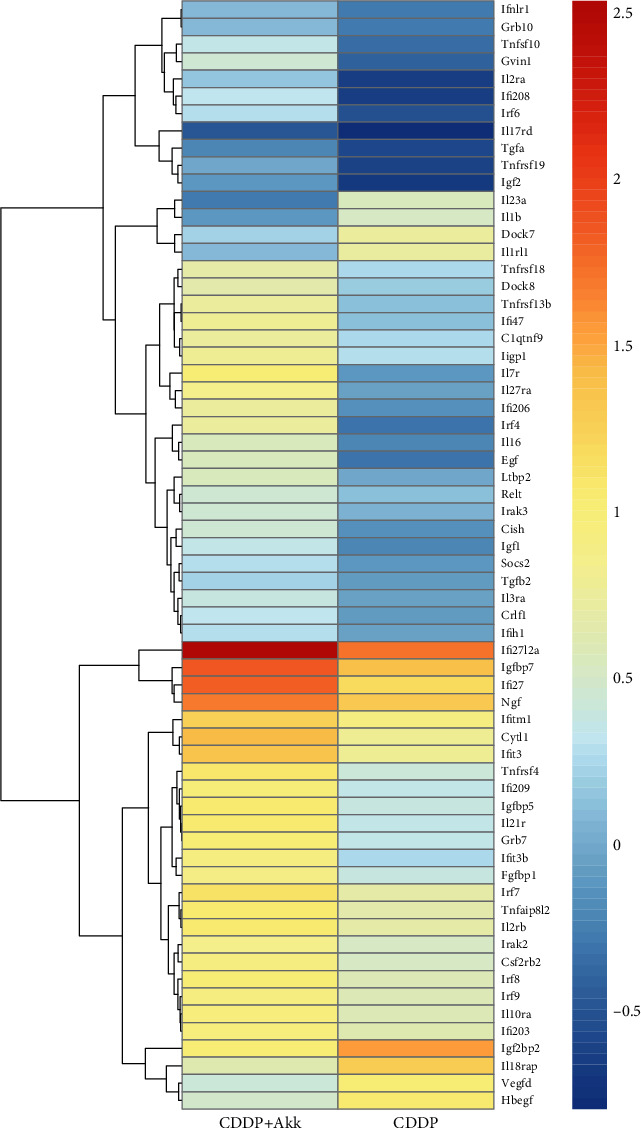
Comparison of immune-related differential genes between the CDDP group and the CDDP+Akk group. The horizontal coordinate is the sample, and the vertical coordinate is the gene. Different colors represent different gene expression levels. The color ranges from blue to white to red, indicating low to high expression levels. High-expression genes are shown in red and low-expression genes are shown in dark blue.

**Figure 4:**
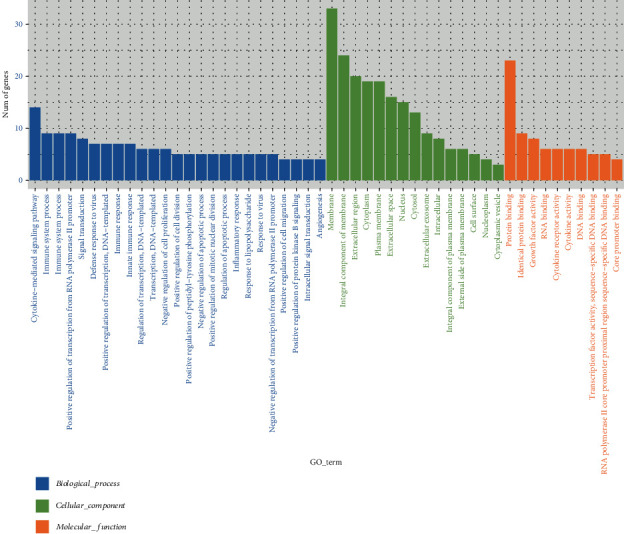
The number and distribution of different genes in GO term.

**Figure 5:**
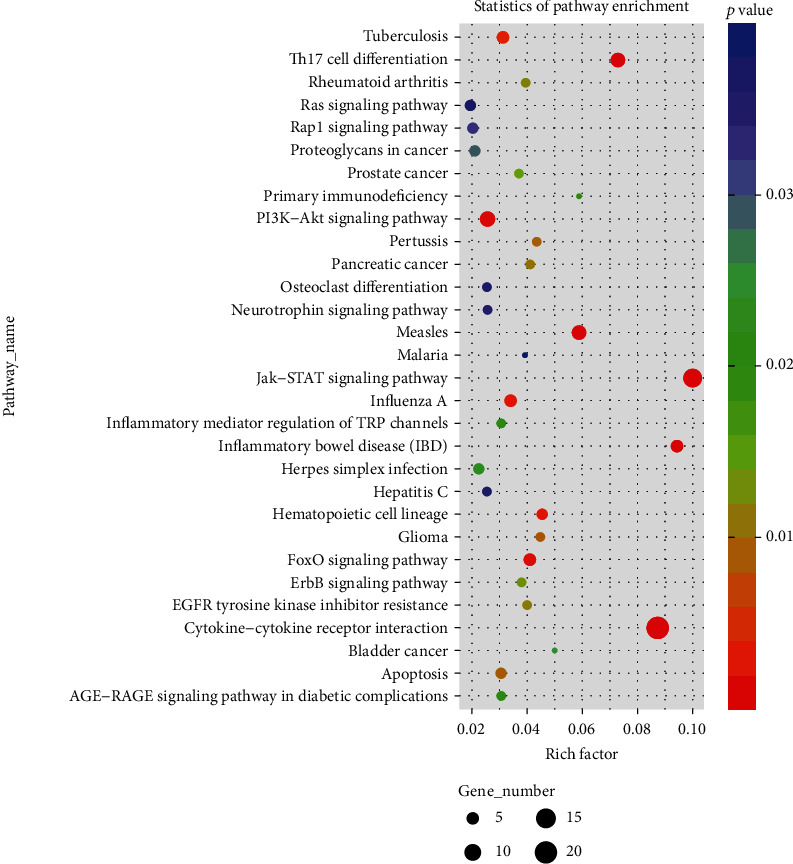
The results of KEGG enrichment analysis. Rich factor represented the number of different genes located in the KEGG/the total number of genes located in the KEGG. The greater the rich factor value was, the greater the degree of KEGG enrichment would be.

As shown in Table 3, 13.56 Gb valid date was obtained after sequencing quality control. The number of reads and the proportion of valid date in the CDDP group were 49873570 (97.16%).The number of reads and the proportion of valid date between the CDDP+Akk group and the reference genome were 37504889 (96.01%).The comparison efficiency of the reference genome selected in this experiment is high, which can meet the needs of subsequent analysis. We conducted cluster analysis of immune-related differential genes according to the similarity degree of the gene expression profile of the sample, so as to visually display the expression of the differential genes. As shown in Figure 3, Ifi27l2a, Igfbp7, Ifi27, and Ngf were highly expressed genes in the CDDP+Akk group. GO analysis was performed on the differentially expressed genes between the CDDP group and the CDDP+Akk group at three stages. As shown in Figure 4, the biological processes involved in differential genes involve cytokine-mediated signaling pathway, immune system process, and regulation of apoptotic process. Cell components related to different genes include the membrane, integral component of the membrane, and the cytoplasm. The molecular functions involved in differential genes include protein binding, growth factor activity, and cytokine receptor activity. The main enrichment pathways of cell immune genetic differences associated with the cytokine-cytokine receptor interaction, JAK-STAT signaling pathway, Th17 cell differentiation, FOXO signaling pathway, and PI3K-Akt signaling pathway are shown (Figure 5).

### 3.4. Effect of Akk on ki-67, p53, Fas, and FasL Proteins Combined with CDDP in Tumor Tissues

**Figure 6:**
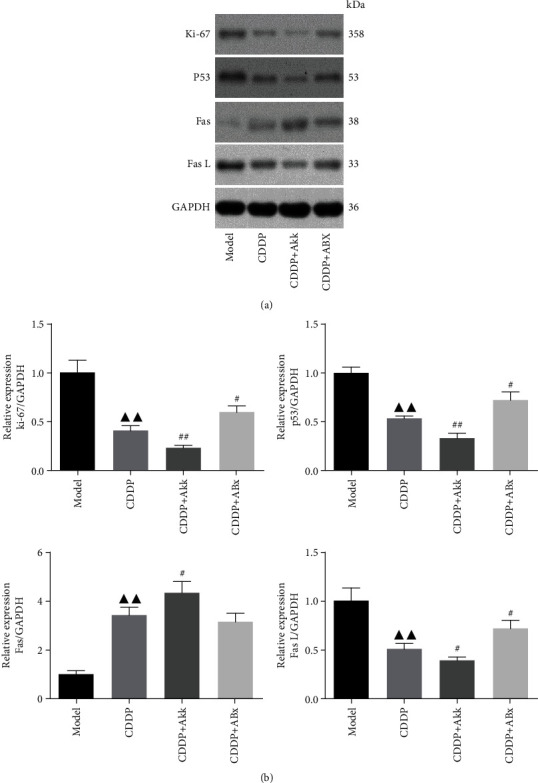
Effect of CDDP combined with Akk on ki-67, p53, Fas, and FasL protein expression in lung cancer mice. The levels of ki-67, p53, Fas, and FasL proteins from tumor tissues were detected by western blot and normalized to GAPDH (a), and then relative band intensities were used in order to quantify ki-67, p53, Fas, and FasL proteins (b). Data were presented as the mean ± SD, *n* = 6 in each group.^▲▲^*P* < 0.01 versus the model group; ^#^*P* < 0.05, ^##^*P* < 0.01 versus the CDDP group.

As shown in Figure 6, tumor-related proteins were detected by western blotting. Compared with the model group, CDDP treatment inhibited the levels of ki-67, p53, and FasL proteins and increased Fas proteins (*P* < 0.01). Compared with the only CDDP group, Akk combining with CDDP aggravated the decrease in ki-67, p53, and FasL proteins and the increase in Fas proteins (*P* < 0.01, *P* < 0.05). The expression levels of ki-67, p53, and FasL proteins in the CDDP+ABx group were slightly increased, while the levels of Fas protein were slightly decreased with no significant difference from the CDDP group.

### 3.5. Effect of Akk on Expression Levels of IFN-*γ*, IL-6, IL-10, and TNF-*α* Combined with CDDP in Mouse Serum

As shown in Table 4, compared with the normal group, the levels of IFN-*γ*, IL-6, and TNF-*α* in the mouse serum of the model group were decreased significantly (*P* < 0.01). Compared with the model group, the levels of IFN-*γ*, IL-6, TNF-*α*, and IL-10 level in the CDDP group were not significantly different. Compared with the model or CDDP group, the serum levels of IFN-*γ*, IL-6, and TNF-*α* in the CDDP+Akk group were significantly increased (*P* < 0.01, *P* < 0.05), while IL-10 level in the CDDP+ABx group was upregulated (*P* < 0.05). There were no obvious difference in the levels of IFN-*γ*, IL-6, and TNF-*α* between the CDDP and CDDP+Akk groups. Compared with the CDDP+ABx group, the serum levels of IFN-*γ*, IL-6, and TNF-*α* in the CDDP+Akk group were increased significantly (*P* < 0.01). IL-10 level was downregulated (*P* < 0.01).

### 3.6. Effect of Akk on Ratio of CD4^+^CD25^+^Foxp3^+^ Treg Cells in Mouse Peripheral Blood and Spleen Mononuclear Cells

**Figure 7:**
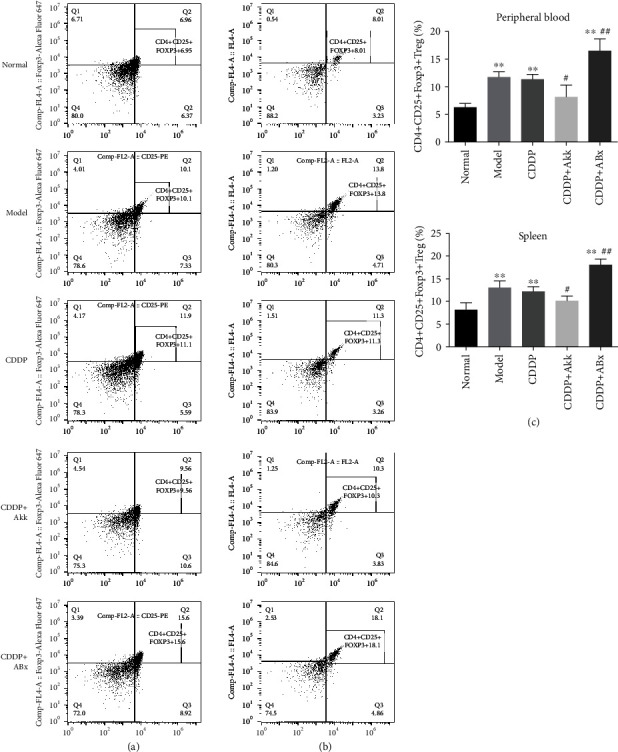
Effect of CDDP combined with Akk on ratio of CD4^+^CD25^+^Foxp3^+^ Treg cells in lung cancer mice. The ratio of CD4^+^CD25^+^Foxp3^+^ Treg cells in mouse peripheral blood (a) and spleen (b) mononuclear cells was measured by flow cytometry, quantified, and plotted in (c). Data were presented as the mean ± SD, *n* = 6 in each group, ^∗^*P* < 0.05, ^∗∗^*P* < 0.01 versus the normal group; ^#^*P* < 0.05, ^##^*P* < 0.01 versus the CDDP group.

As shown in Figure 7, the percentages of CD4^+^CD25^+^Foxp3^+^ Treg cells in the peripheral blood of mice in the model group, CDDP group, and CDDP+ABx group were significantly higher than those in the normal group (*P* < 0.01); there were no obvious difference between the model and CDDP groups. The percentages of CD4^+^CD25^+^Foxp3^+^ Treg in the CDDP group were significantly higher than those in the CDDP+Akk group (*P* < 0.05) and lower than those in the CDDP+ABx group (*P* < 0.01). In total T cells of the spleen, the percentage change trend of CD4^+^CD25^+^Foxp3^+^ Treg cells in each group was the same as that in the peripheral blood group.

## 4. Discussion

Recently, accumulating research evidences uncovered specific intestinal symbiotic bacteria and metabolites can inhibit the occurrence of tumors [14, 15], offering valuable insight into the molecular mechanism of the beneficially immunoregulatory effect of probiotics beyond gut level, which could be applied in prevention or treatment of cancer in extraintestinal sites. Akk, as a next-generation beneficial microbe [16], can modulate gut microbiota composition and intestinal tumor development in mice [17] and influences efficacy of PD-1–based immunotherapy against epithelial tumors [13]. The clinical effectiveness of such chemotherapy is limited by intrinsic or acquired resistance. In this study, the model of mice subcutaneously injected with Lewis lung cancer cells was constructed; then, according to the difference of intraperitoneal injection drugs and feedstuffs, they were divided into the model group, CDDP group, CDDP+Akk group, and CDDP+ABx group. It was found that the antitumor effect was the most obvious in the CDDP+Akk group. With the prolongation of administration time, the inhibition advantage was more obvious, indicating that Akk enhanced antitumor effect in the CDDP treatment of lung cancer. The inhibition effect of CDDP group was followed by that of the CDDP+ABx group. The combination of antibiotics may disrupt the balance of the microflora in mice, so the tumor inhibition rate of mice in the CDDP+ABx group was poor. It was revealed that good symbiotic environment was a necessary condition for the maximum effectiveness of antitumor drugs.

Ki67 is a large nucleolar phosphoprotein and closely correlates with the cell cycle [18]. P53 is a tumor suppressor protein and can regulate the localization, expression, and activity of critical apoptotic effectors. It was reported that median survival time and 1- to 5-year survival rates of non-small-cell lung cancer patients were increased significantly in patients with low expression of Ki67 and p53 (Zhang et al. [19]). In addition, p53 can also induce the expression of Fas and FasL; the induction of which can augment the apoptotic signaling [20, 21]. The binding of FasL to Fas leads to the initiation of death-inducing signal complex, eventually leading to the cleavage of caspase-8 and subsequent release of caspase-3, thus promoting apoptosis [22]. The lower expression of Fas and the overexpression of FasL in non-small-cell lung cancer tissues would lead to immune escape of lung cancer cells (Na et al. [23]). We found that Akk combining with CDDP aggravated the decrease in ki-67, p53, and FasL proteins and the increase in Fas compared with the only CDDP group. The combination of ABx attenuated the downregulation of CDDP on ki-67, p53, and FasL, hinting that the Akk probably participates in the antitumor effect at the gene level through some mechanism, but the direct or indirect effect of the intestinal flora is still to be further explored.

The lung is a mucosal tissue colonized by different bacterial communities. Local microbiota provokes inflammation associated with lung adenocarcinoma by activating lung-resident gamma delta T cells. Germ-free or antibiotic-treated mice were significantly protected from lung cancer development induced by Kras mutation and p53 loss [7]. In vivo, Akk plays a crucial role in maintaining the integrity of the mucus layer, thereby reducing translocation of proinflammatory lipopolysaccharides [24]. Here, our results showed that compared with the CDDP or CDDP+ABx group, Akk combining with CDDP treatment upregulated the levels of IFN-*γ*, IL-6, and TNF-*α* and downregulated IL-10 level. IL-10 in the tumor microenvironment can inhibit the host's antitumor immune response [25]. There was also evidence that Akk exerted anti-inflammatory effects on chronic colitis as they improved clinical parameters and downregulated the expression of the proinflammatory cytokines including TNF-*α* and IFN-*γ* [26]. The interaction between Akk and cytokines may be one of the mechanisms by which Akk plays an antitumor role. Meanwhile, the balance of CD4^+^CD25^+^Foxp3^+^ Treg and CD4^+^IL-17^+^ T17 that are derived from the initial T lymphocyte cells in the tumor microenvironment is disrupted with influence of overexpression of the above inflammatory cytokines. As we all known, the CD4^+^CD25^+^Foxp3^+^ Treg cells can mediate immune tolerance and the CD4^+^IL-17^+^ T17 cells can mediate the inflammatory response, so it is opposite in their functions and differentiation processes, and under normal circumstances, keeping these two cell populations in balance helps maintain immune stability [27, 28]. An increased number of total CD4^+^CD25^+^Foxp3^+^ Treg cells have been observed in the peripheral blood of NSCLC patients [25]. In our study, compared with the CDDP group, combining with Akk treatment suppressed the expression of CD4^+^CD25^+^Foxp3^+^ Treg in the peripheral blood and spleen, but combining with ABx treatment accelerated the expression of CD4^+^CD25^+^Foxp3^+^ Treg, suggesting that Akk could regulate the immune inflammatory microenvironment in the reversion of tumor growth and tumor immune escape.

In addition, a series of bioinformatics methods were used to study the molecular mechanism of the antitumor effect of CDDP combined with Akk on lung cancer. The differentially expressed genes between the CDDP and CDDP+Akk groups from the paracarcinoma lymphoid specimens were screened, and a total of 64 immune-related differentially expressed genes were obtained. Among these differentially expressed genes, IFI27l2 and IGFBP7 were the two most noticeable genes. IFI27l2 (interferon alpha-inducible protein 27-like 2 a) is a member of ISG12 (interferon-stimulated gene protein 12 b) protein family. ISG12 is low level expression within the nuclear envelope under normal circumstances. Type I interferon (IFN) can induce IFI27l2 expression, prompting that ISG12 is positively correlated with the secretion of IFN [29]. In this study, the expression of IFI27l2a was significantly upregulated in the CDDP+Akk group, suggesting that CDDP combined with Akk may induce the production of IFN in the body, which is consistent with the ELISA results. IGFBP7 (insulin-like growth factor binding protein 7) is a member of the insulin-like growth factor binding protein superfamily (IGFBPs), which is mainly synthesized in the liver and exists in multiple organs of the human body [30]. It is currently believed that it may be involved in the development of malignant tumors by regulating cell proliferation, differentiation, and apoptosis. The expression of IGFBP7 in lung cancer tissues is lower than that in normal tissues, so IGFBP7 may be a tumor suppressor gene, inhibiting tumor growth by promoting cell apoptosis and senescence and inhibiting the invasion and migration of cancer cells [31]. We found that the expression of IGFBP7 in the CDDP+Akk group was significantly upregulated, suggesting that CDDP combined with Akk may enhance the antitumor effect of CDDP. We further found that the pathways including the cytokine-cytokine receptor interaction, JAK-STAT signaling pathway, Th17 cell differentiation, FOXO signaling pathway, and PI3K-Akt signaling pathway were associated with antitumor activity of the combination of Akk and CDDP.

## 5. Conclusion

Our findings indicated Akk combining with CDDP slowed down the growth of tumor volume and improved the changes of tumor pathomorphology, downregulated the levels of ki-67, p53, and FasL proteins and upregulated Fas proteins, induced the proinflammatory factor levels such as IFN-*γ*, IL-6, and TNF-*α*, and suppressed the expression of CD4^+^CD25^+^Foxp3^+^ Treg. In addition, Akk combining with CDDP increased the levels of IFI27l2 and IGFBP7 which were the most differentially expressed genes. The antitumor effect of Akk combining with CDDP was related to those pathways including the cytokine-cytokine receptor interaction, Th17 cell differentiation, FOXO, JAK-STAT, and PI3K-Akt signaling pathways. These results suggested that the therapeutic efficacy of the combined treatment of Akk and CDDP was superior to the only CDDP treatment, which could enhance immune regulation and would be a promising strategy for the treatment of lung cancer.

## Figures and Tables

**Table 1:** Body weight of mice (mean ± SD, *n* = 10).

Group	Body weight (g)					
	0 W	1 W	2 W	3 W	4 W	5 W
Normal	24.3 ± 1.7	24.6 ± 1.7	25.0 ± 1.9	25.2 ± 1.9	26.0 ± 1.8	26.1 ± 1.7
Model	25.1 ± 1.9	25.4 ± 1.7	26.8 ± 2.5	25.9 ± 1.5	25.4 ± 1.5	24.6 ± 1.3
CDDP	24.0 ± 1.2	23.6 ± 1.2^▲^	24.1 ± 1.3^▲^	24.0 ± 0.7^▲^	23.5 ± 1.0^▲^	23.2 ± 0.4^▲^
CDDP+Akk	23.9 ± 1.6	23.7 ± 1.9^▲^	24.1 ± 2.6^▲^	24.8 ± 2.5	23.7 ± 1.6	23.5 ± 1.6
CDDP+ABx	24.2 ± 1.4	23.9 ± 1.5^▲^	24.9 ± 2.4	23.9 ± 1.3^▲^	23.2 ± 1.1^▲^	22.8 ± 0.8^▲^

^▲^ *P* < 0.05 versus the model group.

**Table 2:** Volume of tumor in mice (mean ± SD, *n* = 10).

Group	Volume (mm^3^)					
	0 W	1 W	2 W	3 W	4 W	5 W
Model	127.3 ± 37.2	284.4 ± 56.7	1066 ± 253.2	1375 ± 134.4	2106 ± 262.8	2416 ± 396.8
CDDP	124.6 ± 33.0	254.6 ± 49.4	941.5 ± 215.5	1300 ± 369.5	1707.±315.3^▲^	1838 ± 322.4^▲^
CDDP+Akk	140.4 ± 56.3	246.9 ± 64.4	993.1 ± 357.7	1340 ± 336.4	1692 ± 311.5^▲^	1816 ± 233.7^▲▲^
CDDP+ABx	139.5 ± 37.9	270.8 ± 57.3	996.8 ± 349.1	1283 ± 240.8	1831 ± 360.2	2038.0 ± 380.6

^▲^ *P* < 0.05, ^▲▲^*P* < 0.01 versus the model group; ^#^*P* < 0.05, ^##^*P* < 0.01 versus the CDDP group.

**Table 3:** Sequence alignment statistical results between the sample sequencing data and the selected reference genome.

Group	Valid date		Mapped reads
	Reads	Base	
CDDP	51330228	7.70 Gb	49873570 (97.16%)
CDDP+Akk	39063052	5.86 Gb	37504889 (96.01%)

**Table 4:** The levels of IFN-*γ*, IL-6, IL-10, and TNF-*α* in the mouse serum (mean ± SD, *n* = 10).

Group	IFN-*γ* (pg/mL)	IL-6 (pg/mL)	IL-10 (pg/mL)	TNF-*α* (pg/mL)
Normal	426.75 ± 69.17	81.31 ± 12.93	78.85 ± 13.09	526.35 ± 108.11
Model	265.02 ± 59.35	53.22 ± 13.05^∗∗^	81.46 ± 11.71	274.45 ± 67.60^∗∗^
CDDP	244.36 ± 41.92	47.56 ± 7.74	81.04 ± 14.4	260.33 ± 76.93
CDDP+Akk	378.98 ± 76.85^▲##^	78.25 ± 9.17^▲▲##^	62.68 ± 19.07	480.63 ± 96.68^▲▲##^
CDDP+ABx	197.91 ± 44.18	40.12 ± 7.1	108.99 ± 20.4^▲#^	198.05 ± 51.62

^∗^ *P* < 0.05, ^∗∗^*P* < 0.01 versus the normal group; ^▲^*P* < 0.05, ^▲▲^*P* < 0.01 versus the model group; ^#^*P* < 0.05, ^##^*P* < 0.01 versus the CDDP group.

## Data Availability

The raw data supporting the conclusions of this manuscript will be made available by the authors, without undue reservation, to any qualified researcher.
